# Prevalence of Medical Journal Websites That Deny Access to Users Who Block Browser Cookies

**DOI:** 10.1001/jamanetworkopen.2021.3492

**Published:** 2021-03-26

**Authors:** Ari B. Friedman, Evan Miller, Matthew S. McCoy

**Affiliations:** 1 Perelman School of Medicine, University of Pennsylvania, Philadelphia; 2 Leonard David Institute of Health Economics, University of Pennsylvania, Philadelphia; 3 School of Law, Yale University, New Haven, Connecticut

## Abstract

This cross-sectional study of 1700 medical journal websites assesses the prevalence of journals denying access to website users who blocked browser cookies.

## Introduction

Medical journal publishers have recently taken steps to improve public access to research findings.^1,2^ While these changes have been credited with promoting patient empowerment,^3,4^ accessing research related to particular health conditions may carry privacy risks for patients. For-profit companies use website tracking tools to capture information about individuals based on their online activity. These tools raise privacy concerns when they are used on health-related websites where users’ browsing behavior may reveal sensitive information.^5,6^

One step patients can take to protect their privacy when accessing research findings is to adjust their browser settings to block cookies, the data stored on a user’s computer that may be used to identify and track users across multiple websites. However, websites may deny access to users who block cookies, forcing users to choose between accepting privacy risks or foregoing access to content. If medical journal websites employ such practices, it may undermine efforts to improve public access to research findings. We investigated medical journal websites to assess the prevalence of and factors associated with access denial to users blocking browser cookies.

## Methods

This study investigated public websites and therefore was not subject to institutional review board approval because it did not meet the Common Rule’s definition of human subjects research. The study was preregistered on the AsPredicted platform (submission 33300) and followed the Strengthening the Reporting of Observational Studies in Epidemiology (STROBE) reporting guideline for cross-sectional studies.

### Data Collection

We obtained a census of all journals with an impact factor greater than 2.0 in clinically relevant subcategories of the citation research aid Web of Science’s life sciences and biomedicine category. We assessed clinical relevance and identified journals’ open access status, publisher, and website URL address using the protocol described in eMethods in the Supplement.

We used crowdsourcing task website Amazon Mechanical Turk to assess the extent to which journal websites denied access to users blocking cookies. From December 26, 2019, to February 10, 2020, 3 Mechanical Turk workers reviewed the website of each journal included in the study. With browsers set to block cookies, they attempted to access each journal’s homepage, the current issue’s table of contents, and the abstract of a research article. They recorded the level, if any, at which access was denied. Disagreements were resolved by the study team.

### Statistical Analysis

We calculated the percentage and 95% confidence intervals of websites that denied access to users blocking browser cookies, overall and by open access status. Our primary hypothesis was that over 10% of journal websites would deny access to users blocking cookies, assessed by a 2-sided, 1-sample Wilcoxon signed rank test with finite population correction. Our secondary hypothesis was that access denial would be more common among non–open access journals, assessed by a linear probability model with access denial at any level (ie, homepage, table of contents, or abstract) as the dependent variable. Independent variables included open access status, impact factor, publisher, and a categorical variable for publisher size. All hypothesis tests were 2-sided with α = .05. See eMethods in the Supplement for the regression model and sensitivity analyses. Analyses were conducted in R version 3.6.1 (R Project for Statistical Computing).

## Results

Overall, 699 of 1700 journals (41.1% [95% CI, 38.8%-43.5%]) included in the study denied access to users blocking cookies, exceeding our hypothesis (Table 1). Access denial typically occurred at the homepage (eg, of 699 journals denying access overall, 600 [85.8%] were at the level of the homepage and 99 [14.2%] at the table of contents) (Table 1).

**Table 1.** Journal Characteristics Overall and by Open Access Status

Characteristics	Overall, No. (%) (N = 1700)	By access status, No. (%)		*P* value
		Non-Open Access (n = 1454)	Open Access (n = 246)	
Journal impact factor				
2-5	1350 (79.4)	1152 (79.2)	198 (80.5)	.02
5.01-10	253 (14.9)	211 (14.5)	42 (17.1)	
10.01-15	43 (2.5)	37 (2.5)	6 (2.4)	
15.01-224	54 (3.2)	54 (3.71)	0 (0)	
Publisher size^a^				
Small	247 (14.5)	187 (12.9)	60 (24.4)	<.001
Medium	421 (24.8)	290 (19.9)	131 (53.3)	
Top 5	1032 (60.7)	977 (67.2)	55 (22.4)	
Access denial to users blocking cookies	699 (41.1)	651 (44.8)	48 (19.5)	<.001
At homepage	600 (35.3)	554 (38.1)	46 (18.7)	
At table of contents	99 (5.8)	97 (6.7)	2 (0.8)	

^a^ Small publishers were defined as publishers with 10 or less journals, medium publishers as producing between 11 and 86 journals, and top 5 publishers as producing more than 86 journals.

Consistent with our secondary hypothesis, access denial was significantly more common among non–open access journals (651 of 1454 [44.8%]) than open access journals (48 of 246 [19.5%]). After adjustment, non–open access journals were 22.7% (95% CI, 6.3%-39.1%; *P* = .006) more likely to deny access (Table 2). Access policies varied widely among the top 5 publishers. Higher-impact journals did not have significantly different access policies.

**Table 2.** Access Denial Adjusted for Journal Factors

Characteristics	Adjusted % (95% CI)^a^	*P* value
Intercept^b^	8.3 (−6.8 to 23.5)	.28
Publisher size^c^		
Medium	7.7 (−24.4 to 39.8)	.64
Top 5		
Elsevier	7.5 (−3.0 to 18.1)	.16
Lippincott, Williams, and Wilkins	−16.3 (−27.1 to −5.4)	.003
Springer	−30.8 (−41.4 to −20.1)	<.001
Taylor & Francis	69.2 (58.8 to 79.5)	<.001
Wiley	68.5 (58.1 to 79.0)	<.001
Impact factor	0.2 (−0.5 to 0.8)	.63
Non–open access	22.7 (6.3 to 39.1)	.006

^a^ Values represent the results of the linear probability model coefficients and confidence intervals, respectively, multiplied by 100. Results for independent variables (ie, publisher size, impact factor, and non–open access) are interpretable as absolute probability differences from the intercept.

^b^ This regression intercept represents the adjusted access denial percentage for an open access, small-publisher, 0 impact factor journal.

^c^ Medium-sized publishers were defined as publishers producing between 11 and 86 journals, and top 5 publishers as producing more than 86 journals.

## Discussion

This study found that a substantial portion of medical journal websites denied access to users blocking cookies. These findings are noteworthy because they show that taking basic measures to protect one’s online privacy reduces access to research findings. Our study did have limitations, because our search was limited to medical journal websites and did not assess barriers to other online sources of health information.

Many journal websites allow full access regardless of users’ privacy choices. Our findings suggest that journal publishers that currently deny access to users blocking cookies could feasibly modify their websites to allow full access to privacy-conscious users.

## Notes

Supplement. **eMethods.**
