# Supplementary material for: Prevalence of Medical Journal Websites That Deny Access to Users Who Block Browser Cookies
Source: JAMA Netw Open. 2021 Mar 26;4(3):e213492. doi: 10.1001/jamanetworkopen.2021.3492 (PMC7998074; doi:10.1001/jamanetworkopen.2021.3492)
Supplement: Supplement. — eMethods. [file jamanetwopen-e213492-s001.pdf]

Supplemental Online Content

Friedman AB, Miller E, McCoy MS. Prevalence of medical journal websites that deny access to users who block browser cookies. *JAMA Netw Open*. 2021;4(3):e213492. doi:10.1001/jamanetworkopen.2021.3492

eMethods.

This supplemental material has been provided by the authors to give readers additional information about their work.

© 2021 Friedman AB et al. *JAMA Network Open.*

eMethods

Data Collection

Assessing clinical relevance of Web of Science subcategories

Web of Science's Life Sciences and Biomedicine category contains 235 subcategories across health and non-health disciplines. To focus on journals relevant to patients seeking information about their own health conditions, a panel of three clinician-investigators excluded journal subcategories that patients would be unlikely to access for information about their health care.

We considered journals in the following 47 journal subcategories to be medical journals: Allergy, Anesthesiology, Audiology & Speech Pathology, Behavioral Sciences, Cardiac & Cardiovascular Systems, Clinical Neurology, Critical Care Medicine, Dentistry, Oral Surgery & Medicine, Dermatology, Emergency Medicine, Endocrinology & Metabolism, Ergonomics, Gastroenterology & Hepatology, Genetics & Heredity, Geriatrics & Gerontology, Gerontology, Hematology, Infectious Diseases, Integrative & Complementary Med, Medicine, General & Internal, Medicine, Research & Experimental, Nutrition & Dietetics, Obstetrics & Gynecology, Oncology, Ophthalmology, Orthopedics, Otorhinolaryngology, Parasitology, Pathology, Pediatrics, Peripheral Vascular Disease, Primary Health Care, Psychiatry, Psychology, Psychology, Applied, Psychology, Clinical, Psychology, Developmental, Psychology, Experimental, Psychology, Psychoanalysis, Reproductive Biology, Rheumatology, Substance Abuse, Surgery, Transplantation, Tropical Medicine, Urology & Nephrology, Virology

Identifying journal open access status, website URL, and publisher

We determined journals' open access status using the Directory of Open Access Journals.[1]

We used a patient-centered search strategy to determine the URLs of journal websites. For each journal included in the study, 3 Mechanical Turk workers performed a Google search using the search string ("[journal name]" AND "journal") and recorded the URL of the journal homepage. Disagreements were resolved by the study team.

We identified journal publishers, in most cases using journal URLs, which typically included the publisher name. We manually identified publishers of remaining journals by visiting each journal website and recording publisher information.

Data Analysis

There were no missing observations. All hypothesis tests were two-sided with an alpha of 0.05. We assessed for collinearity with Variance Inflation Factor; independent variables were not collinear. We clustered standard errors by publisher. We conducted several sensitivity analyses around the regression results, including model specification (logistic regression), and alternative dependent variables (separate models for homepage, TOC, or abstract access denial). Results did not differ substantively.

Appendix References

1. DOAJ. Directory of Open Access Journals. Accessed March 30, 2020. https://doaj.org

© 2021 Friedman AB et al. *JAMA Network Open.*
